# Inter- and Intra-subject Template-Based Multivariate Synchronization Index Using an Adaptive Threshold for SSVEP-Based BCIs

**DOI:** 10.3389/fnins.2020.00717

**Published:** 2020-09-09

**Authors:** Haoran Wang, Yaoru Sun, Yunxia Li, Shiyi Chen, Wei Zhou

**Affiliations:** ^1^Department of Computer Science and Technolgy, College of Electronic and Information Engineering, Tongji University, Shanghai, China; ^2^Department of Neurology, Shanghai Tongji Hospital, School of Medicine, Tongji University, Shanghai, China; ^3^Department of Information and Communication Engineering, Tongji University, Shanghai, China

**Keywords:** brain-computer interface (BCI), steady-state visually evoked potentials (SSVEP), inter- and intra-subject template-based multivariate synchronization index, transfer learning, adaptive threshold

## Abstract

The steady-state visually evoked potential (SSVEP) has been widely used in brain-computer interfaces (BCIs). Many studies have proved that the Multivariate synchronization index (MSI) is an efficient method for recognizing the frequency components in SSVEP-based BCIs. Despite its success, the recognition accuracy has not been satisfactory because the simplified pre-constructed sine-cosine waves lack abundant features from the real electroencephalogram (EEG) data. Recent advances in addressing this issue have achieved a significant improvement in recognition accuracy by using individual calibration data. In this study, a new extension based on inter- and intra-subject template signals is introduced to improve the performance of the standard MSI method. Through template transfer, inter-subject similarity and variability are employed to enhance the robustness of SSVEP recognition. Additionally, most existed methods for SSVEP recognition utilize a fixed time window (TW) to perform frequency domain analysis, which limits the information transfer rate (ITR) of BCIs. For addressing this problem, a novel adaptive threshold strategy is integrated into the extension of MSI, which uses a dynamic window to extract the temporal features of SSVEPs and recognizes the stimulus frequency based on a pre-set threshold. The pre-set threshold contributes to obtaining an appropriate and shorter signal length for frequency recognition and filtering ignored-invalid trials. The proposed method is evaluated on a 12-class SSVEP dataset recorded from 10 subjects, and the result shows that this achieves higher recognition accuracy and information transfer rate when compared with the CCA, MSI, Multi-set CCA, and Individual Template-based CCA. This paper demonstrates that the proposed method is a promising approach for developing high-speed BCIs.

## 1. Introduction

The Brain-Computer Interfaces (BCIs) provide humans with a direct communication and control channel between human brains and external devices by utilizing brain signals produced along the cerebral cortex within the brain to directly control external devices without the aid of muscular movements (Dornhege et al., 2007; Faller et al., 2010). People with disabilities, such as limb loss, spinal cord injury, and amyotrophic lateral sclerosis, can draw support from BCIs to assist with the activities involved in daily life. Further research is being conducted on developing the EEG-Based Brain-Computer Interfaces due to its non-invasive nature, high temporal resolution, ease of acquisition, and beneficial cost-effectiveness (Nicolas-Alonso and Gomez-Gil, 2012; Al-Hudhud, 2016).

In recent years, several specific brain activity patterns, including Slow Cortical Potentials (SCPs), P300 evoked potentials, Steady-State Visually Evoked Potentials (SSVEPs), Event-Related Desynchronization (ERD), and Synchronization (ERS), have been investigated extensively, as these have served as the source of stimulation signals for BCI control (Zhang et al., 2014b). Among these, the SSVEP paradigm has become a promising option in BCI applications due to its high signal-to-noise ratio (SNR), high information transfer rate (ITR), reliability, and design flexibility (Bin et al., 2009; Zhu et al., 2010; Bakardjian et al., 2011). The SSVEP-BCIs rely on oscillatory responses occurring in the occipital and the occipito-parietal cortex that are elicited from a stimulus flickering at a specific frequency (Vu et al., 2016; Georgiadis et al., 2018). While people focus attention on a visual stimulation at a fixed frequency, such as flashing lights or flickering icons on a computer screen, the SSVEP signals can be observed at the same fundamental frequency as the stimulation and also at higher harmonics of the driving stimulus (Muller-Putz and Pfurtscheller, 2007; Bakardjian et al., 2010; Zhang Z. et al., 2018). Hence, the SSVEP signals are the inherent response of the brain, and the SSVEP-based BCI systems required minimal to no training (Bin et al., 2009).

In the past few decades, many studies have revealed that the SSVEP pattern is effective for BCI control, and various SSVEP-based brain-computer interface (BCI) systems have been proposed by numerous laboratories and research groups (Poryzala and Materka, 2014). It has been verified that four driving rates in an evoked potential interface system are distinguishable (Skidmore and Hill, 1991). In the study, the stimulation frequency was set at 35.050, 23.367, 17.525, and 14.020 Hz, and it was found that the responses corresponding to the stimulation frequencies were generated during the analysis. The SSVEP-based BCI system with high transfer rates was also used to help operators input phone numbers (Cheng et al., 2002) in which four buttons flickering at different frequencies represented the four directions. The operators could move the cursor in different directions to the target position by gazing at these buttons. Finally, eight of the 13 subjects completed the task where subjects were asked to select the correct number on the telephone keypad to input phone numbers with the help of the SSVEP-based BCI system. In another work, a new dual-frequency-SSVEP for BCI systems was developed that could increase the number of selections through different combinations of four frequencies, i.e., 16.4, 17.5, 19.1, and 20.2 Hz (Shyu et al., 2010). The result indicated that this dual-frequency approach was effective for an SSVEP BCI system.

Previous studies for SSVEP recognition focused on the amplitude and spatial distribution of SSVEP responses (Zhang et al., 2013a; Norcia et al., 2015). However, these traditional methods using single-channel EEG data [e.g., Power spectral density analysis (PSDA)] are sensitive to noise and require a long period of recognition time to improve the accuracy of the results. Moreover, these SSVEP recognition techniques cannot detect and identify harmonic stimulation frequencies (Zhang et al., 2011, 2015). Therefore, many advanced multichannel approaches have been developed to enhance the recognition performance of SSVEPs. For frequency recognition, the Canonical Correlation Analysis (CCA) algorithm was first introduced to find the correlation between the multichannel EEG data and reference signals consisting of sin-cosine waves at each of the target frequencies (Lin et al., 2006). Recent work has already validated that the CCA method could achieve better recognition performance than the traditional power spectral density analysis (Zhang et al., 2014c). Until now, there have been many methods proposed to improve recognition accuracy further by optimizing the pre-constructed sine-cosine reference signals, such as Multiway Canonical Correlation Analysis (MCCA) (Zhang et al., 2011), L1-regularized Multiway Canonical Correlation (L1-MCCA) (Zhang et al., 2013b), and Multi-set Canonical Correlation Analysis (Multi-set CCA) (Zhang et al., 2014c)—all proposed as multiway extensions of standard CCA. Although the sine-cosine reference signals usually perform well for specific frequency components recognition, the simplified single or multiple frequency signals are incapable of exactly representing the complex neural responses, which are collaboratively created by several neural populations in the visual cortex rather than a single signal source. Recently, researchers constructed a laminar microcircuits model consisting of two visual areas (V1 and V2) to imitate the dynamics of neuronal population response in the visual cortex, which revealed the modulation mechanism of the SSVEP, confirming the hypothesis (Zhou et al., 2013; Yang et al., 2019). Beside this, the new spatial filtering method, known as Minimum Energy Combination (MEC), found a linear combination of multichannel signals, which reduces the number of channels, to minimize the noise energy (Friman et al., 2007; Nan et al., 2011). Nakanishi et al. used multiple spatial filters to remove the EEG background artifacts, enhance discriminability and SNR of the signals (Nakanishi et al., 2017). Zhang et al. introduced the Correlated Component Analysis (CORCA) to find linear combinations of electrodes across subjects and maximize correlation between them (Zhang et al., 2018a,b). Recently, the Multivariate Synchronization Index (MSI) (Zhang et al., 2014b) has attracted attention as a novel feature extraction method, which calculates the synchronization index between the multichannel EEG data and the pre-constructed reference signals, showing better recognition performance than both CCA and MEC.

Although previous studies have demonstrated that the MSI method is an efficient method for frequency component recognition, the temporal features of the EEG signals have not been explored yet. The analysis of Global Field Power highlighted time periods results in the most robust performance (Jrad and Congedo, 2012), showing the importance of time domain analysis for recognizing the specific frequency in SSVEPs. Recent research has also confirmed that considering temporal information of EEG signals can improve the performance of the algorithm, such as the temporal local structure of the signals (Wang and Zheng, 2008), the time-delayed copy (Lemm et al., 2005), and certain temporal features (Jrad and Congedo, 2012). To address this issue, Zhang et al. proposed a temporally local MSI (TMSI) method, which explicitly considers the time-local information of the EEG signal, further improving the accuracy of the recognition algorithm for SSVEP-Based BCIs (Zhang et al., 2016). The time delay embedding method has also been employed to extend MSI (known as EMSI), further enhancing the performance of SSVEP, which combined the first-order time-delayed version of EEG data during the calculation of the synchronous index (Zhang et al., 2017). Zhang combined adaptive TWL selection strategy with the MSI method, which is superior to fixed TWL in SSVEP recognition (Zhang et al., 2014a).

In the present study, the reference signals of sine-cosine waves are replaced with inter-subject and intra-subject template signals. The intra-subject template signals, also termed as the individual template signals, are obtained by averaging multichannel EEG data of the individual training dataset and provided more abundant subject-specific and inter-trial information for correlation analysis. It has been shown that the CCA based on the individual template signals significantly outperforms the standard CCA (Bin et al., 2011; Nan et al., 2011). Additionally, the inter-subject template signals are obtained by averaging the partial trials selected from other subjects. Recent studies have demonstrated inter-subject similarity in neural responses occurs because subjects are instructed to perform a specific task over time (Saha and Baumert, 2019). Yuan et al. presented transfer template-based canonical correlation analysis (tt-CCA) to enhance the detection of SSVEPs by exploiting inter-subject information (Yuan et al., 2015). Several studies attempted to apply session-to-session and inter-subject transfer to simplify the training procedure (Nakanishi et al., 2016; Waytowich et al., 2016). This paper proposes an efficient way for transfer learning to improve SSVEP-based BCIs performance. After this, an expanding time window over time is used to extract temporal features of SSVEP, and the stimulus frequency is recognized based on the pre-set threshold. Dynamic window recognition algorithms are often integrated into other algorithms to adaptively control the recognition time while maintaining a high accuracy, which significantly improves the information transfer rate (ITR), and adaptability of systems to different individuals (Zhang et al., 2014a; Cao et al., 2015; Yang et al., 2018). In the method presented in this paper, the pre-set threshold obtained from the training dataset of individual subjects makes the algorithm shutdown at the appropriate data length and filters the potentially invalid trial resulted from attention lapses (Russell et al., 2016) or the reaction times of subjects considered to be too long. It has been reported that attention lapses may lead to an increase of reaction times and the number of incorrect responses because irrelevant information cannot be effectively suppressed, shifting attention to irrelevant visual stimuli (Ko et al., 2017; Wang et al., 2018). The novel extension of multivariate synchronization index method is verified with an SSVEP dataset involving 10 healthy subjects and compared to the CCA, standard MSI, Multi-set CCA, and Individual Template-based CCA. The results in this paper show that the proposed method significantly enhances the individual recognition performance of SSVEP frequency, resulting in an improvement in overall accuracy and the information transfer rate.

## 2. Methods

### 2.1. The Standard Multivariate Synchronization Index

The MSI method aims to estimate the synchronization between the multichannel EEG data and the reference signals for frequency detection. Let $X\in {\mathbb{R}}^{N_{1}\times M}$ denote the multivariate EEG signals and $Y\in {\mathbb{R}}^{N_{2}\times M}$ denote the reference signal, which is constructed as follows:

(1) $$\begin{array}{l} Y=\left(\begin{array}{c} \sin\left(2\pi f_{i}t\right)\\ \cos\left(2\pi f_{i}t\right)\\ \vdots \\ \sin\left(2\pi N_{h}f_{i}t\right)\\ \cos\left(2\pi N_{h}f_{i}t\right) \end{array}\right),t=\frac{1}{Fs},\frac{2}{Fs},\ldots ,\frac{M}{Fs} \end{array}$$

where *N*_*h*_ denotes the number of harmonics, *Fs* is the sampling rate. *N*_1_ and *N*_2_ are the number of channels, respectively, and *M* is the number of samples. *X* and *Y* are normalized to have zero mean and unit variance without loss of generality. The covariance matrix of concatenation of *X* and *Y* can subsequently be calculated as

(2) $$\begin{array}{l} C=\left(\begin{array}{cc} C_{11} & C_{12}\\ C_{21} & C_{22} \end{array}\right) \end{array}$$

where

(3) $$\begin{array}{l} C_{11}=\frac{1}{M}XX^{T} \end{array}$$

(4) $$\begin{array}{l} C_{22}=\frac{1}{M}YY^{T} \end{array}$$

(5) $$\begin{array}{l} C_{12}=\frac{1}{M}XY^{T}=C_{21}^{T} \end{array}$$

Because both the autocorrelation and cross-correlation of matrix *C*, which is calculated from the concatenation of *X* and *Y*, could influence the synchronization computing, a linear transformation is employed:

(6) $$\begin{array}{l} U=\left(\begin{array}{cc} C_{11}^{-\frac{1}{2}} & 0\\ 0 & C_{22}^{-\frac{1}{2}} \end{array}\right) \end{array}$$

Then, the transformed correlation matrix can be described as follows:

(7) $$\begin{array}{l} R=UCU^{T} \end{array}$$

Assume λ_1_, λ_2_, …, λ_*P*_ are the eigenvalues of matrix *R*. Then, the normalized eigenvalues are represented by

(8) $$\begin{array}{l} \lambda_{i}^{\prime }=\frac{\lambda_{i}}{\sum_{i=1}^{P}\lambda_{i}}=\frac{\lambda_{i}}{tr\left(R\right)} \end{array}$$

where *P* = *N*_1_ + *N*_2_. Finally, the synchronization index between two multivariate signals can be calculated using the following formula:

(9) $$\begin{array}{l} S=1+\frac{\sum_{i=1}^{P}\lambda_{i}^{\prime }\log\left(\lambda_{i}^{\prime }\right)}{\log\left(P\right)} \end{array}$$

Based on the formula (9), the synchronization index of each frequency *f*_*i*_(*i* = 1, …, *K*) used in SSVEP-based BCI can be calculated. The target frequency *f*_*t*_ can now be computed by the formula.

(10) $$\begin{array}{l} f_{t}=\underset{f_{i}}{\max}S_{i},i=1,\ldots ,K \end{array}$$

### 2.2. Inter- and Intra-subject Template-Based Multivariate Synchronization Index (IIST-MSI)

We propose a variant version of multivariate synchronization index based on transferred inter- and intra-subject template signals. Considering $\chi_{i,h}\in {\mathbb{R}}^{N_{c}\times N_{t}}$, which is the *h*-th trial from the individual training set corresponding to the stimulus frequency *f*_*i*_, an individual template signal $Y_{i}\in {\mathbb{R}}^{N_{c}\times N_{t}}$ is obtained by averaging training trials as

(11) $$\begin{array}{l} Y_{i}=\frac{1}{N_{n}}\overset{N_{n}}{\underset{h=1}{\sum }}\chi_{i,h} \end{array}$$

where *N*_*c*_, *N*_*t*_, and *N*_*n*_ are the numbers of channels, samples, and trials, respectively. For structuring the transferred inter-subject templates, the core issue is how to pick up credible trials. We propose a threshold policy for supervised adaptation of trials. Assume $\chi_{p,i,h}\in {\mathbb{R}}^{N_{c}\times N_{t}}$ is the *h*-th trial recorded from the subject *p* corresponding to the stimulus frequency *f*_*i*_. The confidence of this trial is defined as

(12) $$\begin{array}{l} C_{p,i,h}=\frac{S_{p,i,h}}{\frac{1}{K}\sum_{k=1}^{K}S_{p,k,h}} \end{array}$$

where K is the number of stimulus frequencies, *S*_*p,i,h*_ is the multivariate synchronization index between EEG signals and the sine-cosine reference signals at the labeled stimulus frequency *f*_*i*_, and *S*_*p,k,h*_ is the multivariate synchronization index between EEG signals and the sine-cosine reference signals at the stimulus frequency *f*_*k*_. Only high-confidence trials are selected for transfer learning, and the threshold function for confidence is formulated as

(13) $$f(C_{p,i,h})=\{\begin{array}{cc} \;1, & C_{p,i,h}>1+\ln(\frac{N_{t}}{F_{s}}),\\ -\text{1,} & \;otherwise\text{.} \end{array}$$

where *Fs* is the sampling rate. Suppose *A*_*p,i*_ is a set composed of high-confidence trials belonging to subject *p*, and the initial set is the empty set (*A*_*p,i*_ = ∅). The trial selection procedure establishes an iterator to loop over all trials corresponding to the stimulus frequency *f*_*i*_ and pick up high-confidence trials:

(14) $$A_{p,i}\leftarrow \{\begin{array}{cc} A_{p,i}\cup \{\chi_{p,i,h}\}, & f(C_{p,i,h})>0\\ A_{p\text{,}i}\text{,} & \;otherwise\text{.} \end{array}$$

If *P* is the set of ideal subjects used for templates, the inter-subject template is obtained by averaging high-confidence trials across subjects:

(15) $$\begin{array}{l} Y_{i}^{*}=\frac{1}{\left|P\right|}\underset{p\in P}{\sum }\frac{1}{\left|A_{p,i}\right|}\underset{\chi_{p,i,h}\in A_{p,i}}{\sum }\chi_{p,i,h} \end{array}$$

Then, the sine-cosine reference signals of the standard MSI can be replaced by the inter- and intra-subject template signals. The multivariate synchronization index $S_{i}^{*}$ and *S*_*i*_ between the inter- and intra-subject template signals and the test trial can be calculated with the formula (2–9), respectively. Finally, a sum-of-squares γ_*i*_ based the multivariate synchronization index represents the final detection score for the stimulus frequency *f*_*i*_:

(16) $$\begin{array}{l} \gamma_{i}={\left(S_{i}\right)}^{2}+{\left(S_{i}^{*}\right)}^{2} \end{array}$$

The target frequency *f*_*t*_ can be recognized by the formula:

(17) $$\begin{array}{l} f_{t}=\underset{f_{i}}{\max}\gamma_{i},i=1,\ldots ,K \end{array}$$

### 2.3. Dynamic Window-Based Adaptive Threshold (AT) Strategy

**Figure 1:**
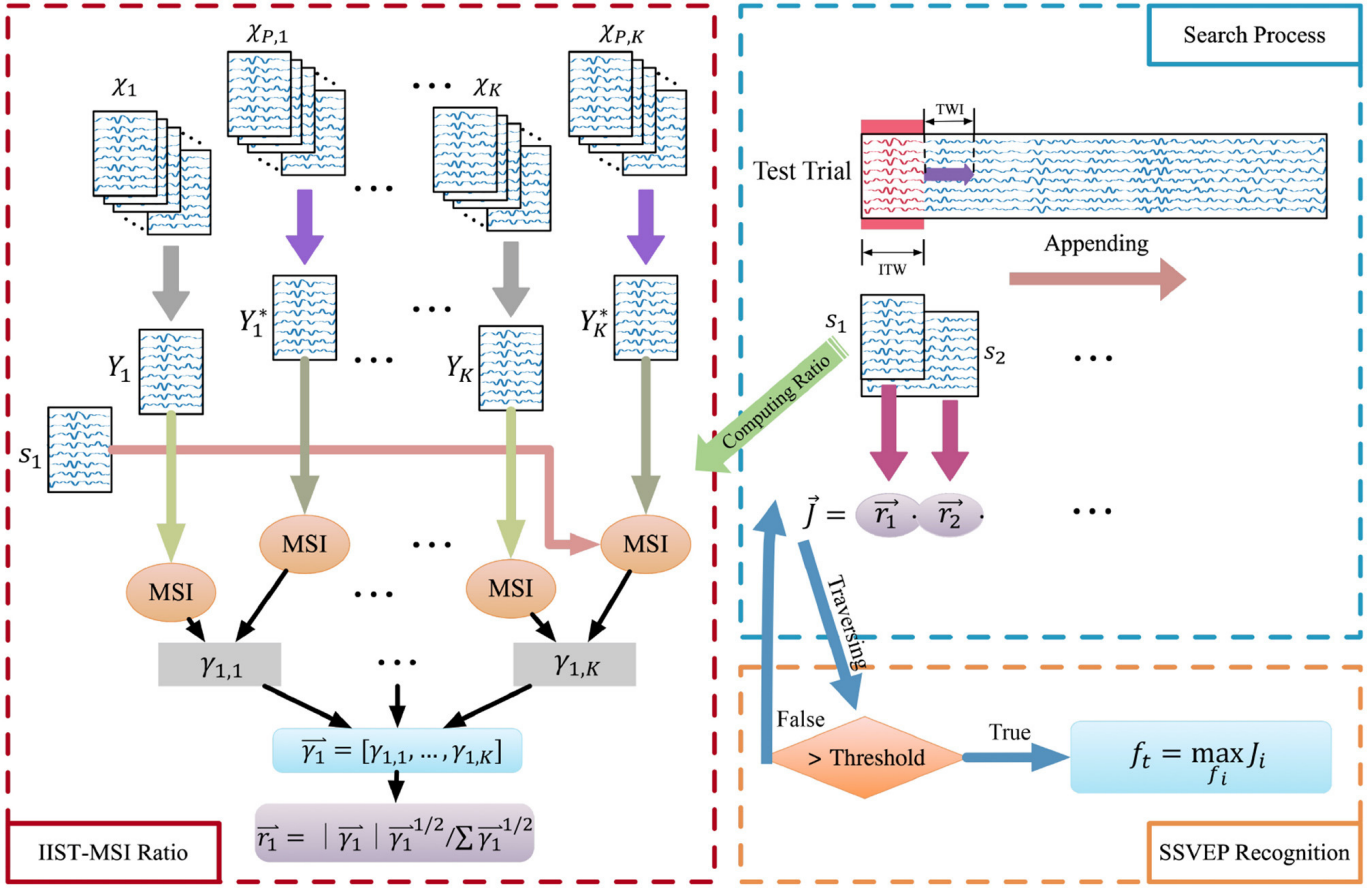
The flowchart of the IIST-MSI-AT method for SSVEP frequency recognition. χ_1_, χ_2_, …, χ_*K*_, and χ_*P*, 1_, χ_*P*, 2_, …, χ_*P, K*_ denote the individual training dataset and that of other selected subjects corresponding to the stimulus frequency *f*_1_, *f*_2_, …, *f*_*K*_, respectively. $Y_{1}^{*},Y_{2}^{*},\ldots ,Y_{K}^{*}$, and *Y*_1_, *Y*_2_, …, *Y*_*K*_ are the inter- and intra-subject templates. Then the synchronization index and the probability ratio of each frequency can be calculated. The probability ratio of each frequency is multiplied, and the result is compared with the threshold. When the threshold is exceeded, the SSVEP frequency can be recognized by the formula (20).

In order to exploit the temporal features of EEG signal, a dynamic window approach is incorporated into the IIST-MSI method. In a trial where the EEG data is continuously received, the inter- and intra-subject template-based multivariate synchronization index of a small initial time window (ITW) corresponding to each stimulus frequency can be first computed. The probability ratio *r*_1, *i*_ of the stimulus frequency *f*_*i*_ can be then defined as

(18) $$\begin{array}{l} r_{1,i}=\frac{\sqrt{\gamma_{1,i}}}{\frac{1}{K}\sum_{k=1}^{K}\sqrt{\gamma_{1,k}}} \end{array}$$

where K is the number of stimulus frequencies. The probability ratio reflects the confidence of each stimulus frequency. When the probability ratio of each stimulus frequency is less than the pre-set threshold, it indicates that the current data length is not enough to make a reasonable decision, so the algorithm requires more data. A time window increment (TWI) is appended to the last data segment, and the algorithm recalculates the probability ratio of this new data segment corresponding to each stimulus frequency. A joint probability of the new data segment and the last data segment can then be computed. After *m* subsequences, the joint probability *J*_*i*_ of the stimulus frequency *f*_*i*_ is calculated as:

(19) $$\begin{array}{l} J_{i}\leftarrow J_{i}\times r_{m,i} \end{array}$$

where the initial value is set as *J*_*i*_ ← *r*_1, *i*_. The threshold *T*_*c*_ serves as the cut-off condition for this method. To paraphrase, if *max*{*J*_1_, …, *J*_*K*_} < *T*_*c*_, the iterative process is continued. When all EEG signals are depleted, and *max*{*J*_1_, …, *J*_*K*_} still is less than *T*_*c*_, the trial is regarded as an invalid trial. Once the method reaches the threshold *T*_*c*_, the target stimulus frequency *f*_*t*_ can be computed as follows:

(20) $$\begin{array}{l} f_{t}=\underset{f_{i}}{\max}J_{i},i=1,\ldots ,K \end{array}$$

where K is the number of stimulus frequencies used in SSVEP-based BCI. Figure 1 illustrates the frequency recognition method.

### 2.4. Contrast Method

For validating effectiveness for frequency recognition in SSVEPs, the classification performance of the proposed method is compared with various algorithms, including Canonical Correlation Analysis (CCA), the standard Multivariate Synchronization Index (MSI), Multi-set CCA, and Individual Template-based CCA.

#### 2.4.1. Canonical Correlation Analysis

Canonical Correlation Analysis (CCA) is a multivariable statistical technique used to reveal the underlying correlation between two multidimensional variables (Hardoon et al., 2004). Given two sets of random variables $X\in {\mathbb{R}}^{N_{1}\times M}$, $Y\in {\mathbb{R}}^{N_{2}\times M}$. Their linear combinations can be define as $\tilde{x}=w^{\text{T}}X$ and $\tilde{y}=v^{\text{T}}X$, respectively. The CCA method is aimed at finding a pair of vectors $w\in {\mathbb{R}}^{N_{1}\times 1}$ and $v\in {\mathbb{R}}^{N_{2}\times 1}$, such that the correlation between $\tilde{x}$ and $\tilde{y}$ is maximized. In other words, the following optimization problem is solved:

(21) $$\begin{array}{l} \rho =\underset{w,v}{\max}\frac{E\left[\tilde{x}\tilde{y}\right]}{\sqrt{E\left[{\tilde{x}}^{2}\right]E\left[{\tilde{y}}^{2}\right]}}=\frac{w^{\text{T}}XY^{\text{T}}v}{w^{\text{T}}XX^{\text{T}}wv^{\text{T}}YY^{\text{T}}v} \end{array}$$

The maximum canonical correlation between the canonical variates $\tilde{x}$ and $\tilde{y}$ is the maximum of ρ. Assume *X* represents a multichannel EEG data, and *Y* is the reference signal constructed according to the formula (1). The maximum canonical correlation of each frequency *f*_*i*_(*i* = 1, …, *K*) can thus be calculated. Then, the target frequency *f*_*t*_ can be recognized by the formula.

(22) $$\begin{array}{l} f_{t}=\underset{f_{i}}{\max}\rho_{i},i=1,\ldots ,K \end{array}$$

#### 2.4.2. Multi-Set Canonical Correlation Analysis

Multi-set canonical correlation analysis (Multi-set CCA) is developed as an extension of CCA to analyze linear relationships between multiple sets of features. In order to improve the classification accuracy of SSVEPs, The Multi-set CCA method is implemented to optimize the reference signal, and the pre-constructed sine-cosine waves, by learning from the joint spatial filtering of training sets of EEG signals (Zhang et al., 2014c).

Assume the *h*-th training trial of EEG signals corresponding to the stimulus frequency *f*_*i*_ is $\chi_{i,h}\in {\mathbb{R}}^{N_{c}\times N_{s}}$, and the spatial filters used to extract common features of training sets are ***w*_1_**, …, ***w*_*n*_**. To maximize the sum of the pairwise correlation between multiple sets of training data, the optimization problem of Multi-set CCA is presented as follows:

(23) $$\begin{array}{l} \begin{array}{c} {\tilde{w}}_{i,1},\ldots ,{\tilde{w}}_{i,n}=\underset{w_{1},\ldots ,w_{n}}{\arg\max}\overset{n}{\underset{h_{1}\neq h_{2}}{\sum }}w_{h_{1}}^{T}\chi_{i,h_{1}}\chi_{i,h_{2}}^{T}w_{h_{2}}\\ \text{subject to}\;\frac{1}{n}\overset{n}{\underset{h_{1}=1}{\sum }}w_{h_{1}}^{T}\chi_{i,h_{1}}\chi_{i,h_{1}}^{T}w_{h_{1}}=1 \end{array} \end{array}$$

The objective function can then be transformed into the following generalized eigenvalue problem with the Lagrange multipliers:

(24) $$\begin{array}{l} \left(R_{i}-S_{i}\right)w=\rho S_{i}w \end{array}$$

where

$$R_{i}=\left[\begin{array}{ccc} \chi_{i,1}\chi_{i,1}^{T} & \ldots & \chi_{i,1}\chi_{i,n}^{T}\\ \vdots & \ddots & \vdots \\ \chi_{i,n}\chi_{i,1}^{T} & \ldots & \chi_{i,n}\chi_{i,n}^{T} \end{array}\right],$$

$$S_{i}=\left[\begin{array}{ccc} \chi_{i,1}\chi_{i,1}^{T} & \ldots & 0\\ \vdots & \ddots & \vdots \\ 0 & \ldots & \chi_{i,n}\chi_{i,n}^{T} \end{array}\right],$$

$$w=\left[\begin{array}{c} w_{1}\\ \vdots \\ w_{n} \end{array}\right]$$

After obtaining the multiple linear transforms ***w*_1_**, …, ***w*_*n*_** and utilizing the joint spatial filtering ${\tilde{z}}_{i,h}={\tilde{w}}_{i,h}^{T}\chi_{i,h}$, the optimized reference signal is constructed as

(25) $$\begin{array}{l} Z_{n}={\left[{\tilde{z}}_{i,1}^{T},{\tilde{z}}_{i,2}^{T},\ldots ,{\tilde{z}}_{i,n}^{T}\right]}^{T} \end{array}$$

Next, the maximum canonical correlation between the test data and the optimized reference signal can be calculated using CCA, and the target stimulus frequency *f*_*t*_ can be recognized with the formula (22).

#### 2.4.3. Individual Template Based CCA

To explore temporal features of EEG signals, the Individual Template-based CCA (IT-CCA) approach was proposed for SSVEP detection (Bin et al., 2011). For each stimulus frequency *f*_*i*_, the individual template signal $Y_{i}\in {\mathbb{R}}^{N_{c}\times N_{t}}$ is obtained by averaging training trials using the formula (11). The CCA process can then be used to calculate the maximum canonical correlation between the test data and the individual template signal, and the target stimulus frequency *f*_*t*_ can be recognized with the formula (22).

### 2.5. Experiment and Data

**Figure 2:**
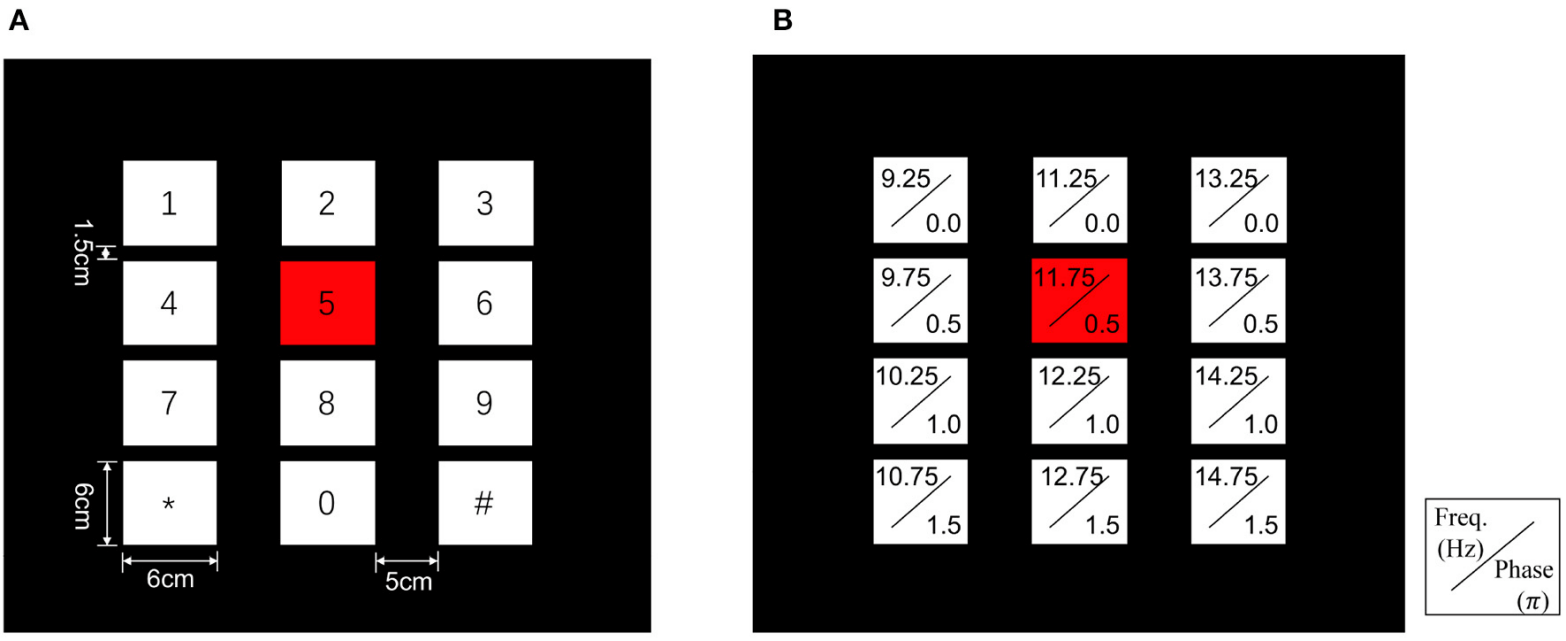
Stimulus design of the 12-target BCI system. **(A)** The user interface of the virtual keypad. **(B)** Frequency and phase values for each target.

To validate our proposed method, a 12-class joint frequency-phase modulated SSVEP dataset from Nakanishi et al. (2015) is used, which contains ten healthy subjects (nine males and one female, the average age being 28 years old), each having 15 trials corresponding to all 12 stimulus frequencies. In their experiment, the 12-target stimuli were presented on an LCD screen with a 60 Hz refresh rate. These stimuli were placed in a 4 × 3 matrix regarded as a virtual keypad, as shown in Figure 2A, and tagged with different frequencies ranging from 9.25 to 14.75 Hz and phases ranging from 0 to 1.5π, as shown in Figure 2B.

When conducting this experiment, the subjects were seated on a comfortable chair within a dim room, with their eyes 60 cm away from the LCD screen. The visual stimuli were presented by the stimulus program in random order. At the beginning of a trial, a red square emerged at the position of the target stimulus for 1 s, which indicated that the subjects should shift their gaze to the target. Afterward, all stimuli started to flicker simultaneously and the subjects were required to stare at the visual stimuli for 4 s. At the same time, EEG signals were recorded with eight electrodes placed over the occipital area with reference to the CMS electrode close to Cz. In this experiment, each subject completed 15 trials corresponding to all 12 targets.

Considering that visual stimulation emerged at the 15th millisecond, the data epochs were extracted from 0.15 to 4.15 s. Each epoch was band-pass filtered from 6 to 80 Hz with an infinite impulse response (IIR) filter and was then used as the input for recognition algorithms.

To determine ideal subjects used for templates, we selected subjects in descending order of the MSI accuracy. For adaptive threshold strategy, the initial time window ITW and the time window increment TWI were set to 0.5 *s*. The threshold *T*_*c*_ took values from a range (*d* ≤ 1*s*: ranging from 1 to 2 with an interval of 0.05; 1*s* < *d* ≤ 2*s*: ranging from 1 to 4 with an interval of 0.1; 2*s* < *d* ≤ 3*s*: ranging from 3 to 8 with an interval of 0.2; and 3*s* < *d* ≤ 4*s*: ranging from 3 to 16 with an interval of 0.4). The number of harmonics pre-defined for reference signals was 3 uniquely. During the process of performing parameter optimization, the combination of parameters would be discarded once the proportion of invalid trials was more than 20%. In the end, an optimum set of parameters was obtained by tuning the parameters to reach maximum recognition accuracy on the training dataset, and the optimal parameters were then applied to frequency recognition of the test dataset.

### 2.6. Evaluation Methods

The classification accuracy is estimated using three-fold cross-validation to evaluate the proposed method. The sample dataset is divided into the training set for choosing the optimal parameter (i.e., the threshold *T*_*c*_) and test set for estimating the performance of the model for frequency recognition. The accuracy is defined as the percentage of valid trials classified correctly. Thus, the classification accuracy is calculated as follows:

(26) $$\begin{array}{l} acc=\frac{1}{3}\overset{3}{\underset{i=1}{\sum }}\frac{P_{i}}{N_{i}}\times 100\% \end{array}$$

where *P*_*i*_ is the number of valid trials correctly classified and *N*_*i*_ is the number of valid trials from the *i*-th fold.

In addition to the classification accuracy, the information transfer rate (ITR) is adopted to evaluate the communication capacity of the BCI system (Wolpaw et al., 2002):

(27) $$\begin{array}{l} B=\log N+P\log P+\left(1-P\right)\log\frac{1-P}{N-1} \end{array}$$

(28) $$\begin{array}{l} ITR=B\times 60/T \end{array}$$

where *P* denotes the classification accuracy, *N* is the number of possible selections, and *T* is the average time required to select a command. Here, the ITR is calculated using different values of *T* (Target gazing time: 0.5 to 4.0 s with an interval of 0.5 s; Gaze shifting time: 1 s).

## 3. Results

**Figure 3:**
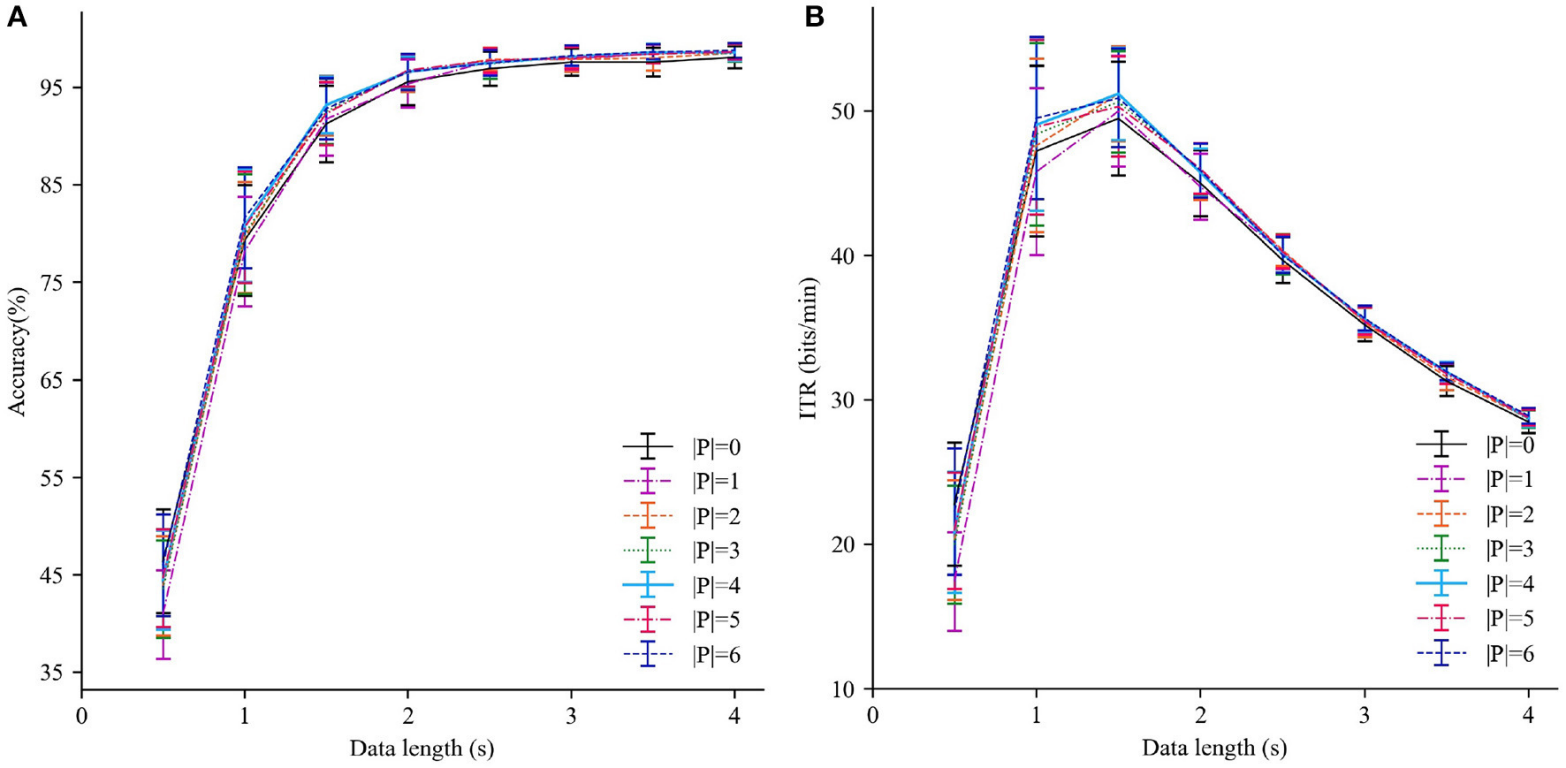
Performance comparison of IIST-MSI with various |*P*|. **(A)** The averaged accuracy and **(B)** ITR across all subjects with different data lengths from 0.5 to 4 s. Error bars show standard errors.

Since the number of subjects used for transfer |*P*| plays an important role in the IIST-MSI method, we explore the effects of varying |*P*| on the recognition performance firstly. As a special case, the individual template-based MSI (IT-MSI) is the same as the IIST-MSI with |*P*| = 0. Figure 3 shows the averaged accuracy and ITR obtained by the IIST-MSI with the |*P*| varying from 0 to 6 and TWs from 0.5 to 4 s. When TW is <1 s, the method only using the individual template performs better than that using the combined inter- and intra-subject templates. When TW is more than 2 s, the result is the contrary. For |*P*| = 4, the IIST-MSI achieved the best recognition performance. In the following analysis, the performance of the IIST-MSI with fixed |*P*| = 4 is compared with that of other methods.

**Figure 4:**
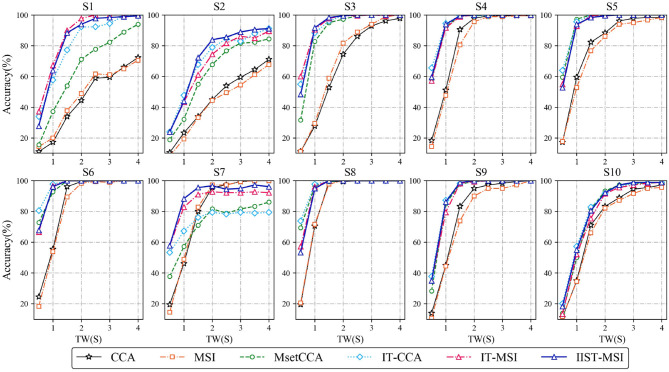
Averaged SSVEP recognition accuracies of 10 subjects derived by CCA, MSI, Multi-set CCA, IT-CCA, IT-MSI, and IIST-MSI, with different length of data epochs from 0.5 to 4 s.

**Figure 5:**
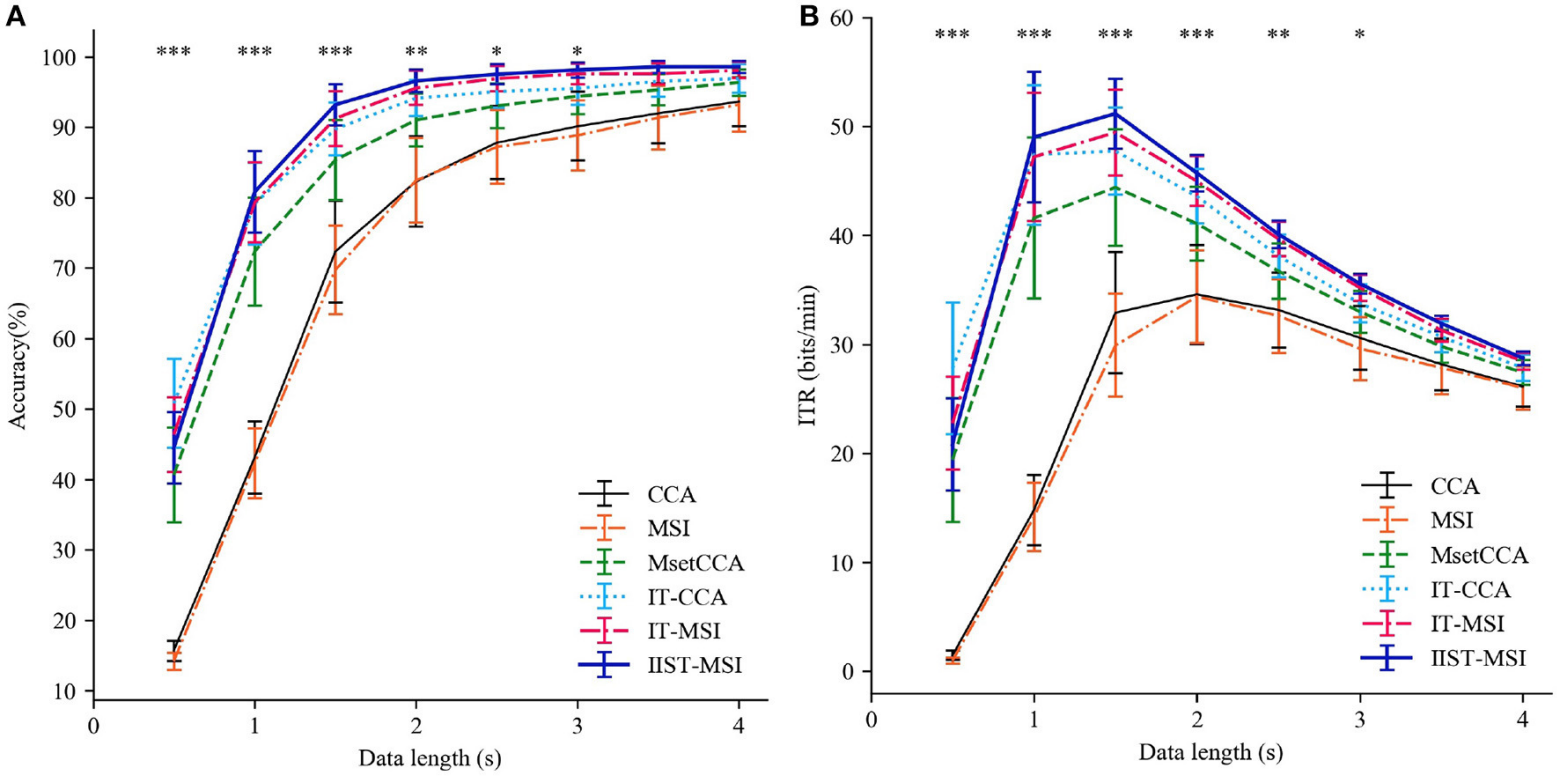
Performance comparison between IIST-MSI and other methods. **(A)** The averaged accuracy and **(B)** ITR across all subjects with different data lengths from 0.5 to 4 s. Error bars show standard errors. The asterisk indicates the statistically significant differences (*p < 0.05; **p < 0.01; ***p < 0.001).

Figure 4 depicts the averaged SSVEP recognition accuracy of ten subjects derived by CCA, MSI, Multi-set CCA, IT-CCA, IT-MSI and IIST-MSI with different data epochs lengths, ranging from 0.5 to 4 s, which shows that the recognition accuracy of subject 2 and 7 is significantly improved by the IIST-MSI. The one-way repeated-measure ANOVA results show that there is a statistically significant difference in the accuracy between these methods under the data length ranging from 0.5 to 3 s [*d* = 0.5 s: *F*_(5, 45)_ = 29.402, *p* < 0.001; *d* = 1*s*: *F*_(5, 45)_ = 52.036, *p* < 0.001; *d* = 1.5*s*: *F*_(5, 45)_ = 11.894, *p* < 0.001; *d* = 2*s*: *F*_(5, 45)_ = 5.269, *p* < 0.01; *d* = 2.5*s*: *F*_(5, 45)_ = 3.395, *p* < 0.05; *d* = 3*s*: *F*_(5, 45)_ = 2.592, *p* < 0.05; *d* = 3.5*s*: *F*_(5, 45)_ = 1.819, 0.1 < *p*; and *d* = 4*s*: *F*_(5, 45)_ = 1.396, 0.1 < *p*]. For a more intuitive comparison of these methods, Figures 5A,B depict the averaged accuracy and the ITR across all subjects with different data lengths from 0.5 to 4 s. In terms of the mean classification accuracies of all ten subjects, from 1 to 4 s, the IIST-MSI method achieves a higher accuracy than the other methods.

**Figure 6:**
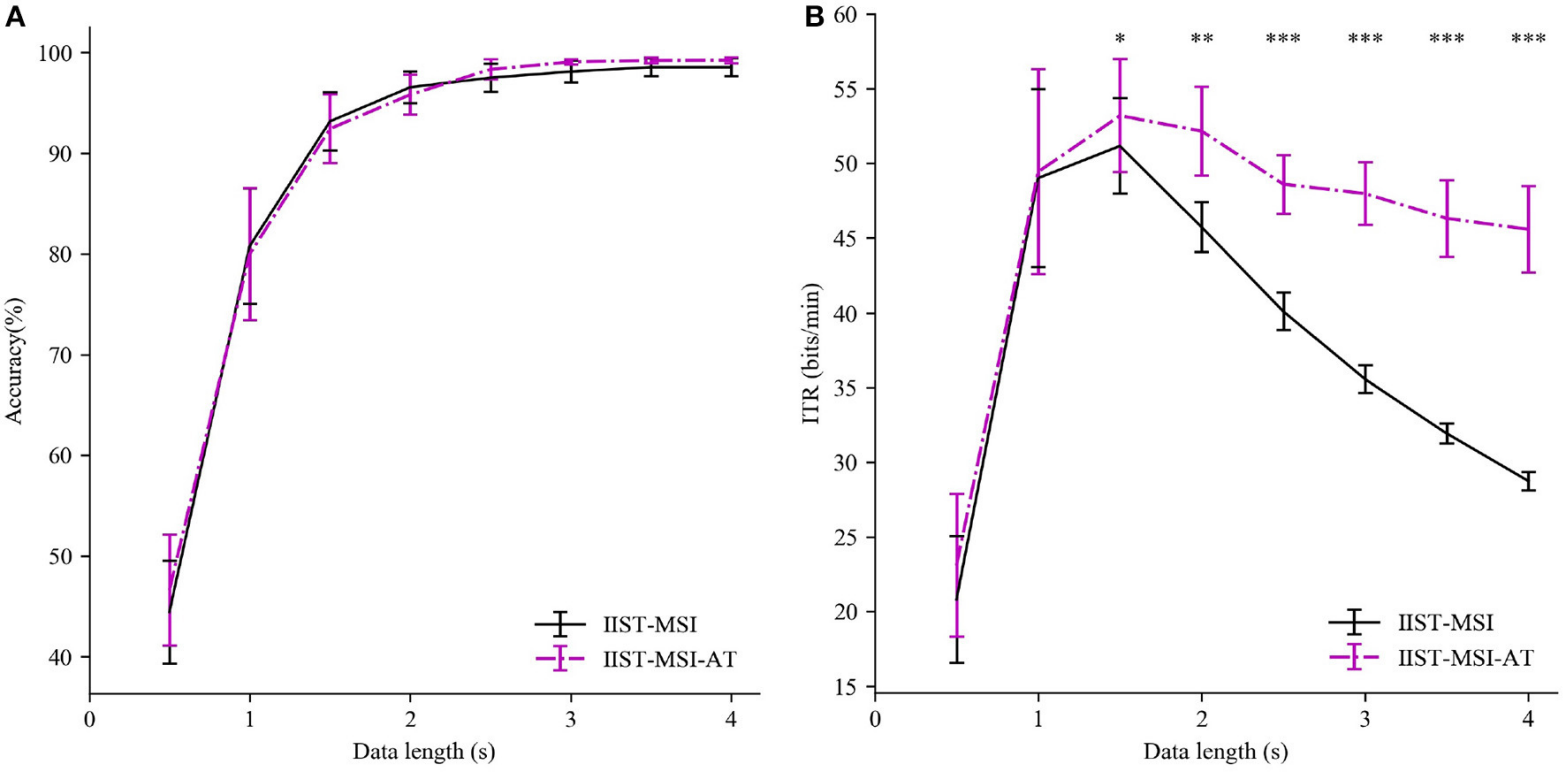
Performance comparison between IIST-MSI and IIST-MSI-AT. **(A)** The averaged accuracy and **(B)** ITR across all subjects with different data lengths from 0.5 to 4 s. Error bars show standard errors. The asterisk indicates the statistically significant differences (paired *t*-tests, *p < 0.05; **p < 0.01; ***p < 0.001).

To investigate the superiority of adaptive threshold strategy, the IIST-MSI using adaptive threshold (IIST-MSI-AT) is compared with the basic IIST-MSI. Figure 6 depicts the mean detection accuracy and ITR for the basic and the combined version of IIST-MSI method. The paired-sample *t*-test shows there are no statistical differences in the accuracy between them, but there are significant differences in the ITR from 1.5 to 4 s. The experimental result coincides with the expectation that the dynamic window algorithm can adaptively determine the shorter time window, while maintaining high accuracy. Hence the IIST-MSI-AT method significantly outperformed the other methods in terms of ITR. The highest ITR obtained by the IIST-MSI-AT method is 53.08 ± 3.65 bits/min.

Table 1 presents the recognition accuracy and ITR obtained by CCA, MSI, Multi-set CCA, IT-CCA, and IIST-MSI-AT for each subject with a 4 s data length. Here, the accuracy of CCA, MSI, Multi-set CCA, and IT-CCA are the average values computed over 180 trials for each subject. The accuracy of IIST-MSI-AT is described as the average accuracy of the test set in a three-fold cross-validation as formulated in the equation (26). For the epoch length of 4 s, the IIST-MSI-AT method gets the highest accuracy (99.23 ± 0.29%),which achieves an increase of 5.62% compared to CCA (93.61 ± 3.48%), 6.06% compared to MSI (93.17 ± 3.82%), 2.90% compared to Multi-set CCA (96.33 ± 1.84%), and 2.34% compared to IT-CCA (96.89 ± 2.02%). These results demonstrate that the proposed method is a promising way to develop more high-performance SSVEP-based brain-computer interface systems.

**Table 1:** Classification accuracy (%) and ITR (bits/min) of CCA, MSI, Multi-set CCA, IT-CCA, and IIST-MSI-AT for each subject with 4*s* data length.

**Subject**	**CCA**		**MSI**		**Multi-set CCA**		**IT-CCA**		**IIST-MSI-AT**	
	**Accuracy**	**ITR**	**Accuracy**	**ITR**	**Accuracy**	**ITR**	**Accuracy**	**ITR**	**Accuracy**	**ITR**
S1	72.22	14.74	70.55	14.07	93.89	25.30	99.44	29.25	98.83	35.12
S2	71.11	14.29	67.78	13.00	84.44	20.16	91.11	23.66	99.32	33.38
S3	97.78	27.90	100.00	29.82	100.00	29.82	100.00	29.82	100.00	52.99
S4	99.44	29.25	99.44	29.25	100.00	29.82	100.00	29.82	100.00	53.04
S5	98.33	28.32	98.33	28.32	100.00	29.82	100.00	29.82	98.89	43.77
S6	100.00	29.82	100.00	29.82	100.00	29.82	100.00	29.82	100.00	57.53
S7	100.00	29.82	100.00	29.82	86.11	20.99	79.44	17.81	97.19	38.43
S8	100.00	29.82	100.00	29.82	100.00	29.82	100.00	29.82	100.00	55.91
S9	100.00	29.82	100.00	29.82	100.00	29.82	100.00	29.82	100.00	51.51
S10	97.22	27.5	95.56	26.36	98.89	28.77	98.89	28.77	98.11	34.17
Mean ± STD	93.61 ± 3.48	26.13 ± 1.85	93.17 ± 3.82	26.01 ± 2.00	96.33 ± 1.84	27.41 ± 1.16	96.89 ± 2.02	27.84 ± 1.2	99.23 ± 0.29	45.58 ± 2.89

## 4. Discussion

The most recent state-of-the-art methods for SSVEP recognition use the individual calibration data as the template of correlation analysis and significantly improve the detection performance (Nakanishi et al., 2015). The individual templates can accumulate the frequency components while maintaining the phase information and, conversely, reduce the effect of the background EEG artifacts. Furthermore, it contributes to improving the individual adaptability of methods, as the individual templates can learn spontaneous EEG signals from calibration data. However, the training data collection process may be time-consuming. The visual fatigue and attention lapses make the training data not perfect enough for every subject. For addressing this problem, inter-subject transfer learning is exploited to provide inter-subject similarity and variability for enhancing target recognition in SSVEP-based BCIs. For each subject, the frequency components of SSVEPs induced by a specific target frequency are similar, but the visual latencies in the visual system are various. According to the superposition principle, the averaged inter-subject transferred templates can contain the same frequency and little phase differences (Yuan et al., 2015). Based on this, this study replaces the commonly used sine-cosine reference signals with the inter- and intra-subject templates for improving adaptability and robustness of the MSI method. Indeed, the experimental results show that the detection accuracy of a few individuals is obviously improved.

On the other hand, this paper employs a dynamic time window to explore the temporal features of SSVEP signals neglected by the standard MSI method and a pre-set threshold to determine when to stop the algorithm, which can balance the recognition accuracy and data length. Hence, the proposed method can significantly improve the information transmission rate, which is critical to the development of high-speed BCIs. Considering the limited reliability of short data, the threshold not only acts as the stopping condition but assists in filtering these invalid trials to avoid wrong commands. Accordingly, the method will improve the effectiveness of the dry-electrode based BCI system with a low signal-to-noise ratio by filtering invalid trials, which can avoid mistakes and ensure the stability of BCI.

## 5. Conclusion

In this paper, we introduce a novel method based on the inter- and intra-subject template and adaptive threshold strategy to enhance the detection of SSVEPs for high-speed BCIs. The experimental results on ten subjects indicate that our approach obtains higher recognition accuracy and ITR than the CCA, MSI, Multi-set CCA, and Individual Template-based CCA. The results remind us that the inter-subject template transfer and the threshold search based on other methods could further improve the performance of BCIs, which will be investigated in our future work.

## Data Availability Statement

Publicly available datasets were analyzed in this study. This data can be found here: https://github.com/mnakanishi/12JFPM_SSVEP.

## Author Contributions

HW contributed conception and realization of algorithm. SC organized the database. WZ performed the statistical analysis. All authors contributed to manuscript revision, read, and approved the submitted version.

## Conflict of Interest

The authors declare that the research was conducted in the absence of any commercial or financial relationships that could be construed as a potential conflict of interest.
